# The Influence of Health Literacy and Self-Management on Emergency Healthcare Access for Rural Asthmatics: A Patient’s Perspective

**DOI:** 10.7759/cureus.50350

**Published:** 2023-12-11

**Authors:** Alannah Stoneley, Judith Anderson, Clare Sutton

**Affiliations:** 1 School of Nursing, Paramedicine and Healthcare Sciences, Charles Sturt University, Bathurst, AUS

**Keywords:** self-management, rural, health literacy, emergency healthcare, asthma

## Abstract

Introduction: Asthma is a leading cause of respiratory illness in Australia and has a significant prevalence in rural areas. Literature suggests living in rural areas can act as a barrier to receiving emergency healthcare for asthma exacerbations resulting in the need for further resources and advocacy in educating rural asthmatics about managing their condition and knowing when to access further help. This study aims to explore decision-making in accessing emergency healthcare for people with moderate to severe asthma exacerbations in rural New South Wales (NSW).

Methods: A qualitative interpretative design involving semi-structured interviews with 12 participants was conducted between May and July 2021. Interviews were recorded and transcribed verbatim. Participants were asthmatics who had accessed emergency healthcare for a moderate to severe asthma exacerbation in NSW over the last five years. Data was thematically analysed and grouped into four overarching themes. Due to the depth and range of data this paper will be reporting on two of those themes.

Results: Past experiences as education and the impact of self-management strategies were both shown to be prominent influences that affected decisions on choosing to access emergency healthcare during a moderate to severe asthma exacerbation. Participants shared how their decision-making was shaped by health literacy gained from their past experiences of asthma exacerbations and dealing with emergency healthcare. In addition, self-management strategies were utilised to minimise the need to access emergency healthcare. This was achieved through telehealth appointments, lifestyle changes and non-pharmacological strategies.

Conclusion: This study shows that an increase in health promotion activities throughout rural areas can help to reassure people about uncertainties that may be associated with their past experiences and improve health literacy. Additionally, providing tools to allow confident self-management of asthma effectively using evidence-based techniques to prevent it progressing to a moderate or severe exacerbation would also be of benefit.

## Introduction

In Australia, asthma is one of the leading causes of respiratory illness amongst all age groups and genders affecting 11% of the population [1]. Ambulance utilisation for an asthma-related emergency has been recorded as suboptimal in rural Australia with an underestimation of the severity of an asthma exacerbation being one of the notable reasons for delayed seeking of emergency healthcare [2-4]. There are a number of studies that demonstrate how the utilisation of education-based programs can assist in the increase in health literacy regarding when to access emergency healthcare for an asthma-related emergency [3,4]. An Australian study focused on the utilisation of asthma action plans found that positive asthma outcomes were associated with both asthma action plans as well as sufficient education into the condition and its management [5]. What is not known is whether there is enough education being provided to allow rural residents to develop the health literacy needed to make appropriate and educated decisions regarding emergency asthma management. Therefore it is important to investigate areas that may benefit from education-based programs to increase the health literacy of residents, thus improving emergency asthma care. This study aims to do this by exploring the attitudes and behaviours of rural asthmatics towards accessing emergency healthcare for moderate to severe asthma exacerbations in rural New South Wales (NSW). This paper illustrates the health literacy that rural NSW asthmatics were utilising to inform their decision-making in accessing emergency healthcare both through ambulance services, emergency departments and implementing self-management strategies. This information allows us to formulate an educated opinion on whether there is enough access to both equitable emergency healthcare and health literacy in these areas.

## Materials and methods

To meet the aim of representing the patient’s perspective, this study followed a constructivist paradigm. Ontologically this acknowledges that although a physical reality exists there are multiple perspectives on the social construction of that reality. Epistemologically the researcher is situated as interacting with the data and the subjective nature of the perspectives being represented. Thematic analysis aligns with this framework and allowed insight into the overall interpretation of understanding how participants make decisions regarding their healthcare [6].

The research questions focussed on: 1. What influences rural and remote asthma patients to call/not call for an ambulance during an exacerbation? and 2. What are the experiences of asthma patients in utilising emergency healthcare in rural and remote NSW? 

This paper discussed the data uncovered from the first research question and the overarching themes that came from this. A qualitative interpretative design was utilised involving the collection of data through semi-structured interviews. To study the phenomena of interest, recruitment targeted people who had accessed emergency healthcare in rural NSW due to a moderate to severe asthma exacerbation within the last five years. Recruitment of participants was achieved through purposive sampling based on the inclusion criteria outlined in Table 1. 

**Table 1:** Inclusion Criteria NSW: New South Wales *Modified Monash Model [7]

Criteria	Description
Age	Participants needed to be a minimum of 18 years old.
Severity	Participants needed to have accessed emergency healthcare for asthma with an exacerbation of moderate to severe intensity.
Time	This event must have occurred within the last 5 years to ensure participants could accurately recall the events and emotions from their experiences.
Location	Only participants whose event occurred in postcodes which were located in NSW and scored an MM3 or above on the Modified Monash Model* were included in this study
Other	The study was open to people who lived in these areas or who were travelling through them when the exacerbation occurred and was open to the patient themselves, or family members or carers who were involved in the decision regarding when to access emergency healthcare.

Recruitment occurred through advertisements over social media platforms and interested people meeting the inclusion criteria (Table 1) were provided with an information sheet and consent form when registering their interest. Participants engaged in a semi-structured interview via Zoom. Participants were asked to discuss what influenced them when deciding to access emergency healthcare and prompt questions were used to encourage participants to elaborate and provide examples. As part of the constructivist paradigm, participants were encouraged to express what was most important to them and what they would want healthcare workers to understand about their experiences. Interviews lasted approximately one hour, and the recordings were transcribed verbatim by a paid organisation. Participants were assigned pseudonyms and the transcripts were deidentified prior to analysis. Participants were provided with a debrief statement at the end of the interview containing support services in case of distress caused by telling their stories, however no participants reported feeling distressed. Analysis took place concurrently with interviewing and interviewing ceased when no new themes were identified in the final two interviews - an indication that data saturation had been reached [8].

The data was initially analysed (managed with NVivo) to identify codes that seemed significant to the topic or had been identified by participants as being important. These codes were then combined into common themes which captured the important concepts from the data set. All researchers discussed the emerging themes, and an audit trail was kept to track their development and monitor preconceptions and reflexivity [9]. Further refinement and defining of each theme were achieved through discussion by all researchers until consensus was reached following Braun and Clarke’s method of thematic analysis [10,11]. Four overarching themes were developed from the responses to the research question. 

Ethical considerations were addressed, with confidentiality of information assured. Participants all indicated they were pleased to have the opportunity to tell their stories through both written and verbal consent. This project was granted ethics approval by the Charles Sturt University Human Research Ethics Committee (approval number H21044).

## Results

Details of the 12 participants and their geographical location are summarised in Table 2. Four overarching themes were identified from the 12 interviews. Themes included the impact of healthcare logistics, past experiences as education, the impact of self-management strategies and the cost of accessing emergency healthcare. Due to the range and the depth of data, this paper discussed the results from two overarching themes: past experiences as education and the impact of self-management strategies. 

**Table 2:** Participant details *Modified Monash Model [7]

Participant name: (pseudonym)	Modified Monash number*	Approximate age (nearest 10 years)
Suzie	MM4	40
Tracey	MM3	50+
Jessica	MM5	30
Mel	MM3	30
Kylie	MM4	30
Kevin	MM3	50+
Joseph	MM5	50+
Briony	MM3	50+
Jason	MM5	50+
Lisa	MM3	40
Jen	MM4	30
Cecilia	MM3	50+

Past experiences as education

Participants discussed how their past experiences provided them with education that impacted their decision to access emergency healthcare. They articulated their decision-making was shaped by health literacy gained through these past experiences. Participants also noted that their past, present, and future actions were motivated by their experiences of accessing emergency healthcare, by the negative attitudes of healthcare workers and by their overall confidence in the emergency healthcare services accessed. Furthermore, they perceived that healthcare staff deemed their condition was less serious when they self-presented to an Emergency Department (ED) rather than arriving via ambulance which impacted on their decision-making. 

Impact of Health Literacy

Health literacy influenced participant’s decisions on accessing emergency healthcare. When participants didn’t understand the role of paramedics in stabilising or treating asthma exacerbations, they were more inclined to self-present to the ED. 

For example, Joseph explained his preference to self-present to the ED because he was seeking more definitive treatment. Whilst he predicted paramedics could treat his asthma exacerbation on the spot, he lacked information on paramedic referral pathways or their role in liaising with medical staff at handover to ensure the need for additional support or ongoing monitoring.

“I wasn’t sure what they would have had on board. I knew they probably could have given me something in the short term, but I was looking for something I could hold in my hand and take home, but I knew that they {paramedics} could fix me on the spot.” (Joseph)

Similarly, Tracey shared not knowing what paramedic treatment would involve, motivated her to self-present to the ED. She stated that being unsure of a paramedic’s scope of practice led to deciding to bypass the ambulance and make her own way to hospital.

“Yeah, I don’t know. I’ve never had the experience of calling an ambulance with an asthma attack, so I don’t know what their {paramedic} treatment will be.” (Tracey)

Overall, participants’ limitations in health literacy regarding utilising ambulance resources to treat asthma exacerbations impacted their decision-making when accessing emergency healthcare. Participants learned to self-present to ED rather than calling for an ambulance, repeating their historical behaviours due to a lack of health literacy regarding paramedic treatment. 

History of Asthma

It was also found that participants with a longer history of living with asthma felt they had better education based on their past experiences, allowing them to gauge the severity of their asthma. They felt this enabled them to more accurately determine whether they needed to access emergency healthcare for their asthma or if they would be able to self-manage it. 

This was illustrated by Lisa when explaining the factors affecting her decision-making in relation to her child’s asthma. 

“I think we’ve just been dealing with it for a very long time and know when to go and when to wait a little longer.” (Lisa) 

Furthermore, Briony articulated the advantages of knowing her body as she had been living with the asthma for many years. She explained that through her past experiences of living with asthma and exacerbations she felt confident in her decision-making regarding when and how to access emergency healthcare and being able to recognise deterioration that would cause her to change her decision.

“That’s been a real turning point for me with my asthma over the last few years … I know my body better than anybody else and being confident in knowing that this is what’s happening … that makes a big difference.” (Briony) 

Participants with a history of asthma were able to use the learning gained from their past experiences to influence their decisions on how to self-manage their asthma as well as how and when they should access emergency healthcare. This knowledge could act as a useful tool in giving participants additional time to access emergency healthcare services when they need to but was also influenced by the attitude of healthcare workers. 

Negative Healthcare Worker Attitudes

Participants discussed how attitudes they were faced with when accessing emergency healthcare noticeably influenced what they would do when they experienced further exacerbations. Participants reported that sometimes when they would self-present to the ED, they would be faced with negative attitudes from healthcare staff implying they did not need to be there. These attitudes influenced participants’ decisions on accessing emergency healthcare in the future. 

Cecilia discussed how her experiences of accessing emergency healthcare were characterised by staff not listening to her, and suggesting they knew best. She used the example where healthcare staff made multiple attempts to cannulate her even after she told them she was difficult to cannulate. 

“I suppose it doesn’t matter what knowledge you have. It’s the knowledge of the doctor, and the nurses that are dealing with you that affects how you get treated, because they don’t give a shit whether you were an ex-nurse, or you’ve been asthmatic since you were 7. I get the impression often that ‘I don’t know what’s best for me’ from them … I have terrible veins. Even anaesthetists can’t sometimes canulate me and they all just don’t believe me. And then the seventh canulation attempt later, they get out the ultrasound machine.” (Cecilia)

Cecilia described another experience where she had self-presented to the ED and had not received immediate treatment despite not being able to talk due to breathlessness (a significant feature indicating the need for urgent treatment). Despite this, Cecilia indicated her preference was still to self-present to the ED if she had further exacerbations as she did not want to waste paramedic resources.

“There’s been times I’ve walked in, and I have not been able to talk, and I’m still shoved in a chair and no other thing {treatment} and it’s just sort of, ‘Oh well can you sit over here? You’ll be right’ type of thing ... I’ve worked in a lot of A&Es {accident and emergency}. And I’ve always seen that people used ambulances to skip the queue I suppose. If an ambulance comes in, you get priority and I know a lot of my friends are like this. We just don’t want to be seen wasting resources” (Cecilia)

Lisa explained times when she had presented to the ED due to her daughter’s asthma exacerbations and the attitude of healthcare staff suggested they felt it was unnecessary, even though it was recommended in her daughter’s asthma management plan. Lisa said she preferred to present to the ED rather than calling an ambulance, but the attitudes of staff made her hesitant to do so.

“It’s not all the time, but a lot of the time if we present with her walking, breathing, talking, {and} she’s not blue, we get looked at like, ‘What’s wrong with her? Why have you brought her up here?’ They don’t quite understand … when she’s really struggling.” (Lisa)

Similarly, Jen’s perception was that if she presented to the ED by ambulance, she would be seen faster and was more likely to be admitted. This may be due to having a healthcare worker (paramedic) advocating for her during handover to the ED. In comparison she believed that healthcare staff appeared less concerned about her condition when she had been able to self-present. These perceptions impacted Jen’s decision on how to access emergency healthcare.

“I think if I present via ambulance, they’re more likely to see me faster and possibly even admit me whereas if I self-present I feel like they kind of go, ‘Well you got yourself here, so you’re probably not that bad’.” (Jen)

Overall, participants highlighted the impact of negative healthcare worker attitudes on how comfortable they felt accessing emergency healthcare. Ultimately, despite these negative attitudes, participants stated they would still access emergency healthcare when they needed it, however in some cases they may ‘think twice’ or wait a little longer based on these past experiences. In Cecilia’s situation, even though she perceived she would be given lower priority if she self-presented, there were other factors motivating her to continue to self-present to the ED. However, in Lisa and Jen’s cases, they were more likely to call for an ambulance in the future to be assessed faster. These attitudes were concerning as a delay in seeking emergency healthcare could have a significant impact on the outcomes experienced by participants due to the nature of moderate to severe asthma exacerbations. Furthermore, negative healthcare worker attitudes also impacted participant’s confidence in emergency healthcare.

Confidence in Emergency Healthcare

The confidence participants had in emergency healthcare also influenced their decision on when and how to access it. Confidence related to the faith participants had in the ability of the healthcare workforce to provide effective and timely treatment. Participants discussed confidence placed in hospital-based care, sometimes comparing the care they received in rural NSW with care they received in the city. Participants also addressed confidence placed in pre-hospital care when describing influences on their decision-making to access emergency healthcare.

Briony recognised the need for experience to ensure that a health professional was competent in providing care. She recognised that in her small local hospital, there were unlikely to be many patients like her presenting which may impact the quality of care she would receive; however, she expressed confidence that her local hospital was able to identify her need for treatment and knew when to transfer her to a hospital with more resources. This confidence made it easier for Briony to make the decision to access emergency healthcare when it was needed, even though she acknowledged that her local rural health service was not as well-resourced as those in larger communities. 

“In a rural and remote area, we get a lot of less experienced nurses, and less experienced doctors … When I’ve had to go to the hospital in the city, they immediately recognise {my condition}, and it’s triaged well. So, I think it’s probably just lack of experience and because you’re not going to get a severe asthma attack coming in every day … The good thing is, they {rural healthcare workers} know if you have to go somewhere else, they’re quick to transfer you.” (Briony)

Cecilia on the other hand felt that the relationship she had built with healthcare professionals in her small community ensured that they were aware of her medical history and were more effective in treating her than their counterparts in the city. She was particularly satisfied with the personal care she felt would only be received in such small communities providing her with confidence in accessing it.

“In the rural situation, you know who the ambos are… it feels almost like a personal service... In the city I think you never know who’s going to turn up … you lose a little bit of that personal touch in the city, and I think that makes a big difference. The same with the hospital, it sounds strange, but I pretty much know everyone that works in ER… often they’ll say, alright which problem are you in for today? What’s happening is it the (medical condition A)? Is it the asthma?” (Cecilia) 

Furthermore, when considering pre-hospital care, Jen was confident that the paramedics in her rural location would be able to manage her exacerbation, however, she worried they may not be able to reach her in a timely manner.

“I feel like they’d {paramedics} be able to manage it, I would be concerned that they wouldn’t get there in time, that’d be my concern.” (Jen)

Overall participants were confident the healthcare workers in the hospital environment could stabilise their initial presentation, however they also discussed the need to be transferred to larger hospitals with better resources as a common occurrence, particularly for severe exacerbations. Although participants felt there was a substantial difference between healthcare provided in rural and metropolitan areas, they disagreed about the impact it had on their level of satisfaction. Additionally, participants had confidence that paramedics would be able to manage their initial asthma exacerbation however, they had fears that paramedics may not be able to reach them in good time.

In conclusion, past experiences and health literacy had an impact by educating participants on their decision-making regarding when and how to access emergency healthcare. Participants demonstrated a lack of health literacy about paramedic asthma management which affected their decision-making on accessing pre-hospital emergency healthcare. Additionally, participants past experiences and confidence also impacted how and when they would access emergency healthcare in the future. Many participants identified that they also had developed skills to manage their own care.

Self-management strategies

Many participants utilised self-management strategies to minimise the need to access emergency healthcare. This included utilising telehealth appointments as well as other strategies which helped participants to manage moderate to severe asthma exacerbations at home or minimise their occurrence. The implementation and effectiveness of these methods influenced the participant’s decision to access emergency healthcare. 

Telehealth

One way participants were able to manage their asthma, decreasing the need to access emergency healthcare, was through telehealth appointments with asthma specialists. This method helped to overcome accessibility issues in rural NSW allowing participants to easily connect with specialised healthcare professionals. This improved asthma management, decreasing the incidence of moderate to severe exacerbations and reducing the need to access emergency healthcare. 

When describing her experiences utilising telehealth for her asthma, Briony shared it was beneficial in increasing access to healthcare services to manage her asthma effectively.

“I think it’s trying to think outside the square so to speak on how else we can access health and services and keep things up to date.” (Briony)

Suzie expressed how telehealth benefited her asthma management as her specialist was able to monitor her breathing through this platform. She explained how this was important during the COVID-19 pandemic as she would otherwise have struggled to maintain adequate contact with her specialist. 

“Because my specialist is in (MM3 town), apart from having to go down to do a breathing test, I’ve been able to do all my other appointments via telehealth … and she can still monitor my breathing … I am a chronic asthmatic, and I chose to stay at work during COVID, so they were touching base quite frequently with me just to make sure that I was on top of my medications.” (Suzie, lived in MM4 town).

Overall, telehealth proved to be beneficial in keeping participants connected with asthma specialists in different areas without traveling long distances which was frequently identified as an issue for rural healthcare. Participants who had taken advantage of this platform found it beneficial as it provided better access to services, they needed to manage their asthma effectively to decrease the occurrence of exacerbations and avoid the need to access emergency healthcare. Other participants made lifestyle changes to better manage their overall health.

Lifestyle

Other self-management strategies that participants utilised were alterations to their daily lifestyle to better manage their asthma. Strategies included removing themselves from situations that were known to trigger exacerbations, and alterations to diet and activity to improve their overall health.

Kevin explained he was trying to be more active and adopt a healthier diet to improve his health and better manage his asthma independently. 

“A little bit of dietary stuff and trying to get back into my exercise. So before being diagnosed I used to run every day, and now I can’t run every day, so we walk every day. Eventually I want to be able to run again and dietary {changes} like more water intake, reduced salt intake, trying to control cholesterol.” (Kevin) 

Cecilia discussed the main triggers which caused her asthma to be exacerbated. With this knowledge she altered her lifestyle so that she did not expose herself to these triggers. 

“So, we get a lot of dust storms out here. And I know if there’s a dust storm, I don’t go out in it. I pressurise the house, lock it all up. Even at work I basically say, no if there’s a dust storm, I’m not going out on {outside duties}. You need to find some other way to do that, because one of my big triggers is the dust. And we find that about 4 days post dust storm that’s when I always have to present at hospital.” (Cecilia) 

Overall, participants in this study described several ways that they altered their daily activities to gain better control of their asthma to prevent the occurrence of exacerbations. When discussing their decision to access emergency healthcare, they identified how a change in lifestyle or other strategies could play an important part in their need to not access emergency healthcare. 

Other Strategies

Participants also described several nonpharmacological self-management strategies to effectively manage moderate to severe asthma exacerbations. Strategies included inhalation of essential oils to help control breathing during exacerbations. Other participants described how they utilised household items such as fans to create a breeze in the face and aid in the administration of Ventolin. 

Briony explained how essential oils inhaled through a humidifier had supported her to manage asthma exacerbations.

“For me it’s about finding alternatives you know, like I have essential oils, so I use {brand name} essential oils and they’ve got a formula called ‘breathe’ which helps with breathing and stuff like that and keep the airways clear.” (Briony) 

Furthermore, Kylie reported that throughout her experiences of managing her daughter’s asthma she found a breeze in her face in addition to the use of Ventolin helped to manage asthma exacerbations.

“The only thing that we have found that works well is when she is having an attack and it’s quite bad is, we just put her in front of the fan. Apparently, that just helps them get their breath and breathe a bit better … making sure that she does get that Ventolin in her.” (Kylie)

Overall, some participants showed a preference for utilising self-management strategies to manage asthma exacerbations rather than accessing assistance from healthcare professionals. Although this was admirable and made them feel that they were in control of their situation, there were some circumstances where these self-management strategies could have been improved with advice from healthcare professionals, particularly as the participants in this study were discussing moderate to severe asthma management.

In conclusion, participants had acquired several self-management strategies to aid in preventing and managing exacerbations. Services such as telehealth appointments with asthma specialists as well as tools and strategies to improve overall health were ways in which participants better managed their asthma on an everyday level to both prevent the occurrence of exacerbations and attempt to avoid the need to seek emergency healthcare. It was also important to note that these strategies could also be utilised whilst waiting to receive emergency healthcare via ambulance or on the way to self-present to the ED, however some were not evidence-based and may have delayed more effective treatment.

## Discussion

The aim of this study was to explore the influences on rural asthmatics in calling or not calling for an ambulance during a moderate to severe asthma exacerbation as well as exploring their experiences in utilising emergency healthcare in rural and remote NSW. This project identified several themes with the focus of this paper being the influence of past experiences as education and the impact of self-management strategies. This research allowed us to explore the current health literacy of NSW asthmatics and identify factors that inform their decision-making on how to access emergency healthcare in the event of a moderate to severe asthma exacerbation. 

The theme past experiences as education showed the impact of health literacy as well as personal experiences associated with having a history of asthma influenced participants decision-making when accessing emergency healthcare. However not having optimal health literacy can be classified as a barrier in accessing emergency healthcare and is outlined in Morgans et al.'s study [2], which uncovered gaps in the health literacy of Australian rural asthmatics when accessing emergency healthcare for an asthma emergency. Overall, their study found suboptimal health literacy led to misunderstandings of the ability of paramedics to aid, creating barriers to understanding when it is appropriate to seek help [2]. Similarly, other Australian studies such as Boyd and Archer [3] and Larson et al. [4] highlighted a limited understanding of asthma and its seriousness in rural Australia but show promising results of education-based programs leading to an improvement in asthma-related health literacy. More recently, Denford, et al.'s [12] exploration of psychological and communication processes which occur with asthma intervention found that prior to appropriate intervention, asthma patients misconceived their condition and in some cases did not recognise that they had asthma. Participants in this study revealed a lack of knowledge on when it was appropriate to call for an ambulance or what paramedic treatment may include indicating the potential need for further asthma and emergency healthcare education in these areas. 

Furthermore, it could be assumed that the influence of health literacy and previous history of asthma has a greater influence on rural residents’ decision-making due to the barriers associated with access to resources which is prevalent in these areas. Chen and Tescher [13] share the pressure placed on rural Australian EDs because of low numbers of specialists and government funding in addition to serving large geographical locations. Likewise, Pate et al. [14] noted rurality was associated with poorer health outcomes and related this to limited access to resources due to shortages in the healthcare workforce and higher prevalence of hospital closures which resulted in longer distances being travelled to access emergency healthcare. Both can also influence confidence in emergency healthcare as conditions can deteriorate markedly due to limited staff and lesser resources and can also be seen through experiences shared by participants in this study.

Additionally, the impact of self-management strategies influenced participants' decision to access emergency healthcare. Primarily this was utilised by participants in attempt to prevent and manage asthma-related emergencies without having to seek any external emergency healthcare support. Due to the limited access to resources and increased travel times to receive emergency healthcare, participants discussed how telehealth has been implemented in asthma-related management. Telehealth has become more prominent over recent years; Kew and Cates [15] suggested this may reduce health inequalities in rural areas, however, they could not clearly determine whether at-home telehealth monitoring impacted the need to access emergency healthcare. More recently, Locke et al. [16] found that telehealth could be a useful tool in improving inhaler technique and medication compliance in rural asthmatics, however, once again this could not be linked with a difference in asthma-related hospitalisations. Those participants in this study who had utilised telehealth for their asthma found it beneficial as it allowed them to connect with asthma specialists they would otherwise have trouble accessing, further emphasising the benefits of telehealth in general asthma management. However, this study did not review direct links between the benefits of telehealth with asthma emergency healthcare. 

Changes in lifestyle such as modifications to diet and exercise and other strategies including the utilisation of essential oils were responses that influenced how participants accessed emergency healthcare by choosing to make simple day-to-day changes in their lifestyle and develop self-management strategies to manage moderate to severe asthma exacerbations. Cvetkovski et al. [17] saw Australians confidently creating their own self-management strategies to manage their asthma at home due to their limited healthcare access. This was seen to both directly and indirectly affect participants’ management of asthma emergencies. In the current study some changes to lifestyle were implemented in attempts to reduce exacerbations whilst other interventions were created to self-manage exacerbations as the first response before accessing emergency healthcare similar to the behaviours outlined in Cvetkovski et al. [17] which were self-management through the use of patients own medication devices and written asthma information. However, unlike Cvetkovski et al. [17], not all self-management strategies in this study were evidence-based suggesting it is important to be cautious of these behaviours as Fennell et al. [18] described how certain behaviours, such as the need to have self-reliance and control, can be a barrier to seeking help for health issues amongst rural Australians. 

Both past experiences as education and the impact of self-management strategies were interconnected and ultimately had a significant influence on the decision-making of rural NSW asthmatics in accessing emergency healthcare for a moderate to severe asthma exacerbation. It was identified that their self-management strategies and changes to lifestyle were influenced by their health literacy which they had gained mainly from their history of asthma and past experiences of accessing emergency healthcare. Moving forward, knowing the influence of these experiences, health promotion policies can be implemented in these rural areas to ensure that these populations are making informed decisions on accessing emergency healthcare during asthma-related emergencies based on current and appropriate educational material.

Limitations

A limitation of this study was that participants were recruited from one state only. This study was limited to NSW to provide a greater depth of insight as people with asthma in other states may have different experiences of rural health (e.g., scope of practice, availability of services) and comparison across different healthcare systems would not have been possible within the constraints of this study. This topic would benefit from a larger-scale study covering multiple Australian states and healthcare services. This study was limited to a small number of participants, but addresses criteria of trustworthiness, including credibility (where the research team all checked the results and analysis); transferability (where the participant quotes provide a depth of information that allows the results to be transferred to similar contexts), dependability (including a description of how the analysis proceeded), and confirmability (where the direct quotes provided demonstrate clearly how the themes were developed) [9]. Despite these limitations, data saturation was still able to be reached with limited participants and this has allowed a focused understanding to be developed of the geographical location studied to add valuable recommendations to this environment. 

## Conclusions

The findings of this research show that there is a heavy reliance on using past experiences as education when accessing emergency healthcare for an asthma exacerbation. Participants demonstrated a lack of health literacy regarding paramedic asthma management, impacting on their decision-making in accessing pre-hospital emergency healthcare. However, services such as telehealth appointments with asthma specialists and other strategies to improve lifestyles were believed by participants to have positive effects. It is important to note that not all strategies were evidence-based and not all participants utilised the self-management strategies discussed. This exploration highlights the significant impact that levels of education and health literacy have on asthmatics in rural NSW and how they can benefit from linking in with services such as telehealth and educational programs to help prevent exacerbations or to manage exacerbations more effectively until they reach definitive care. This study shows that an increase in health promotion activities throughout regional areas could help to reassure people about uncertainties that may be associated with their past experiences and improve health literacy in addition to providing tools to be able to confidently self-manage mild asthma effectively using evidence-based techniques to prevent it from progressing to a moderate or severe exacerbation.
